# RBMS1 promotes gastric cancer metastasis through autocrine IL-6/JAK2/STAT3 signaling

**DOI:** 10.1038/s41419-022-04747-3

**Published:** 2022-03-31

**Authors:** Mengyuan Liu, Heming Li, Huijing Zhang, Huan Zhou, Taiwei Jiao, Mingliang Feng, Fangjian Na, Mingjun Sun, Mingfang Zhao, Lei Xue, Lu Xu

**Affiliations:** 1 Department of Gastroenterology, The First Hospital of China Medical University, 110001 Shenyang, China; 2 Department of Endoscopy, The First Hospital of China Medical University, 110001 Shenyang, China; 3 Department of Medical Oncology, The First Hospital of China Medical University, 110001 Shenyang, China; 4 Network Information Center, China Medical University, 110122 Shenyang, China; 5 Department of Oral and Maxillofacial Surgery, School of Stomatology, China Medical University, 110001 Shenyang, China

**Keywords:** RNAi, Experimental models of disease

## Abstract

Metastasis is the most important reason for the poor prognosis of gastric cancer (GC) patients, and the mechanism urgently needs to be clarified. Here, we explored a prognostic model for the estimation of tumor-associated mortality in GC patients and revealed the RNA-binding protein RBMS1 as a candidate promoter gene for GC metastasis by analyzing GOBO and Oncomine high-throughput sequencing datasets for 408 GC patients. Additionally, RBMS1 was observed with overexpression in 85 GC patient clinical specimens by IHC staining and further be verified its role in GC metastasis via inducing EMT process both in in vitro and in vivo experiments. Moreover, we identified that IL-6 was predicted to be one of the most significant upstream cytokines in the RBMS1 overexpression gene set based on the Ingenuity Pathway Analysis (IPA) algorithm. Most importantly, we also revealed that RBMS1 could promote migration and invasion through IL6 transactivation and JAK2/STAT3 downstream signaling pathway activation by influencing histone modification in the promoter regions after binding with the transcription factor MYC in the HGC-27 and SGC-7901 GC cell lines. Hence, we shed light on the potential molecular mechanisms of RBMS1 in the promotion of GC metastasis, which suggests that RBMS1 may be a potential therapeutic target for GC patients.

## Introduction

Gastric cancer (GC) is one of the most common malignant diseases and a leading cause of cancer death worldwide; moreover, its mortality rate is increasing [1]. Although the clinical outcomes of early GC have improved due to early detection and curative resection, the prognosis of the advanced disease remains poor. Most advanced GC patients die because of tumor recurrence or metastasis [2, 3]. Unfortunately, the mechanisms involved in GC metastasis have not been fully clarified, which hampers the development of anticancer therapy. Hence, it is necessary to explore effective metastatic biomarkers and potential therapeutic targets to improve the long-term survival of advanced GC patients.

GC metastasis is a biological cascade that requires a multitude of genetic interactions [2]. Research progress on the GC metastatic mechanism has provided valuable knowledge for explaining this complex process [4]. Nevertheless, several fundamental questions concerning the mechanisms of GC metastasis remain unclear due to the complexity and biological nature of this process. MYC is an oncogene involved in cell cycle regulation, cell growth arrest, and cell adhesion function in GC [5–7]. A member of the MYC single-strand binding protein (MSSP) family, RNA-binding motif, single-stranded-interacting protein (RBMS) contributes to the regulation of DNA replication, transcription, apoptosis, and cell cycle progression by interacting with the c-Myc protein [8, 9]. In mammals, the RBMS family consists of 3 members termed RBMS1, RBMS2, and RBMS3. It has been reported that RBMS3 plays a tumor suppressor role by downregulating c-Myc and β-catenin [10]; low expression of RBMS3 predicts a poor prognosis in patients with GC and esophageal squamous cell carcinoma [11, 12]. However, the function and regulation of RBMS1 in GC and its relationship with clinicopathological features remain to be elucidated. The potential involvement of RBMS1 in GC metastasis and the related mechanisms still need to be investigated.

As the most prevalent mechanism of GC metastasis, immune regulation has been shown to play important roles in this process and has been widely studied in recent years [13]. IL-6 is a pleiotropic cytokine involved in immune regulation that interacts with IL-6R and activates the Janus kinase/signal transducer and activator of transcription 3 (JAK/STAT3) downstream signaling pathway [14]. Multiple studies have reported that IL-6 is involved in the progression and metastasis of a variety of tumors, including cervical, prostate, lung, breast, and ovarian cancers, through autocrine secretion [15–19]. Most importantly, higher serum IL-6 levels are an independent predictor of a poor prognosis in GC; GC cells can secrete IL-6 and promote tumor growth, development, and migration [20, 21]. Nevertheless, the definite role of the autocrine loop of IL-6 in RBMS1-mediated GC metastasis has not yet been studied.

In this study, we found that RBMS1 predicts poor clinical outcomes in GC based on publicly available databases and GC tissue samples from our clinical cancer center. Furthermore, we provided new insights into RBMS1-mediated GC metastasis for the first time and explored the molecular mechanism underlying GC metastasis: RBMS1 promotes GC metastasis as a core gene through the autocrine IL-6/JAK2/STAT3 signaling pathway via cytokine transactivation by influencing histone modification in promoter regions.

## Materials and methods

### In silico analysis

Gene expression levels were normalized as log2 values in the GeneSpring software (Agilent Technologies, Palo Alto, CA, USA). Genes that were upregulated or downregulated with a greater than twofold change in the RBMS1 overexpression group compared with the vector control group were collected. We further performed computational simulation by using Ingenuity Pathway Analysis (IPA; QIAGEN, Valencia, CA, USA) online tools to predict potential upstream regulators and canonical pathways. Pathway analysis was performed with the genes and proteins with a >2.0-fold change in expression and an activation *z* score of over 2.0 compared with vector control cells identified from the microarray and proteomics data.

### Cell lines, culture conditions, and cell transfection

The GC cell lines MGC-803, BGC-823, and SGC-7901 were kindly provided by Dr. Liu (China Medical University), and other GC cell lines were purchased from Shanghai Institute of Biochemistry and Cell Biology, Chinese Academy of Sciences (Shanghai, China). All these GC cell lines had no mycoplasma contamination and were grown in RPMI-1640 medium (Gibco; Thermo Fisher Scientific) containing 10% fetal bovine serum (FBS), penicillin (10 U/mL), and streptomycin (100 mg/mL) in a humidified atmosphere of 5% CO_2_ at 37 °C. Cells showing a viability >98% were used for experiments.

### Construction of stable RBMS1 knockdown or overexpression cell lines

To obtain stable cells with RBMS1 knockdown or overexpression, RBMS1-RNAi lentiviral vectors and human RBMS1 vectors were constructed by GeneChem Co., Ltd. (Shanghai, China) using the vectors GV248 and GV287, respectively. Then, specific lentiviruses (namely, LV-RBMS1 shRNA and LV-RBMS1) were generated and harvested after 293T cells were transfected with the vector and cultured for 48 h. To establish the stable cell line, the RBMS1-RNAi lentivirus was transfected into SGC-7901 and HGC-27 cells at a multiplicity of infection (MOI) of 10. After 72 h, the transfection efficiency was observed through a fluorescence microscope (BX61, Olympus, Japan), and RBMS1 expression was determined through Western blot analysis. Cells transfected with lentivirus with empty vectors were used as controls.

### Cell viability assays

The cell suspension containing HGC-27 cells was cultured on a 96-well plate at a concentration of 2 × 10^5^/mL, and that containing SGC-7901 cells was cultured at a concentration of 5 × 10^4^/mL. The above cells were divided into 4 groups consisting of a blank group, a negative control group, and experimental groups with knockdown or overexpression of RBMS1. Cell-free medium alone was used for the blank group. Each group was tested three times. After the specified incubation time (0, 24, 48, and 72 h), 20 µL of MTT solution (5 mg/mL) was added to each well, and the mixture was gently shaken and incubated at 37 °C and 5% CO_2_ for 4 h. Then, each well was aspirated with a vacuum aspirator, and 200 µL of dimethyl sulfoxide (DMSO) solution was added. The 96-well plate was shaken for 10 min on a horizontal shaker. The OD value at 570 nm was measured using a microplate reader, and cell viability was calculated according to the following formula: relative cell activity = (OD570 measurement − OD570 blank)/(OD570 control − OD570 blank) × 100%.

### Migration and invasion assays

Cell migration was measured using Transwell chambers with 8.0-µm pore size membranes (Corning, New York, NY). GC cells transfected with lentivirus were seeded into each upper chamber, and 500 μL of the medium in 2.5% FBS was added to each lower chamber of 24-well culture dishes. After 24 h of incubation, the non-migrated cells in the upper chamber were carefully removed with a cotton swab. Then, the migrated cells on the outer side of the membrane were fixed with 4% formaldehyde for 15 min and stained with a 0.1% Giemsa stain solution. The number of migrated cells was counted in five different fields and imaged under a microscope at ×20 magnification.

The cell invasion assay was performed using Matrigel invasion chambers (BD Biosciences). A total of 50 µg of 10% Matrigel (Corning Inc., New York, NY, USA) was used to coat each upper chamber. GC cells that were transfected with siRNA in a serum-free medium were added to each upper chamber. RPMI 1640 supplemented with 10% FBS was added to each lower chamber. After incubation for 48 h at 37 °C, the membrane facing the lower chamber containing invaded cells was gently removed and mounted on a glass slide. The subsequent steps for fixation, staining, and enumeration of cell numbers were the same as those described above.

For interleukin-6 (IL-6) or IL-6 antibody treatment, cells were cultured in a medium containing 200 ng/mL IL-6 (R&D Systems, MN, USA) or 400 µg/mL IL-6 neutralizing antibody (R&D Systems, MN, USA).

### Wound healing assays

The scratch wound healing assay was performed 24 h after cell transfection. HGC-27 and SGC-7901 cells were seeded in a six-well plate until they were confluent, and a 200-μL pipette tip was used to scrape a straight line. Then, the cells were incubated with a fresh medium without FBS for 24 h. Images were captured 0 and 24 h after scratching. ImageJ software was used to analyze and quantify the blank area. All experiments were repeated three times, and statistical analysis was conducted.

### Western blot assay

Total protein was extracted using radioimmunoprecipitation assay lysis buffer (Beyotime Biotechnology, Nanjing, China) according to the manufacturer’s instructions. Then, the extracted protein was mixed with 3× loading buffer and boiled at 95 °C for 5 min. Western blotting was performed as described in our previous study [10]. In brief, the samples were subjected to electrophoresis in an 8% SDS-polypropylene gel at a concentration of 30–50 µg/lane for 3 h and then transferred to a nitrocellulose membrane. After blocking with 5% skim milk for 1 h, the transfer membrane was cut according to the molecular weight of the prestained marker. The membrane was incubated with the indicated primary and secondary antibodies, and the proteins were visualized by an enhanced ECL kit (Beyotime Biotechnology, Nanjing, China). GAPDH was tested as a loading control in the same sample panel. The densitometric results were analyzed with the ImageJ software (Bethesda, MD, USA). The primary and secondary antibodies are listed in Table S1.

### Total RNA extraction and real-time PCR

Total cellular RNA was extracted using TRIzol (Invitrogen, Carlsbad, CA, USA) according to the TRIzol reagent instructions, and all operations were performed on ice. RNA was extracted and reverse transcribed into cDNA according to the PrimeScript® RT Reagent Kit with gDNA Eraser (Takara, Japan) protocol. The SYBR^®^ Premix Ex TaqTM II method was used to detect relative mRNA expression. GAPDH was used as the internal reference. The primer sequences were as follows: RBMS1: forward, 5’-AAGGTCACTAAGCAGCACAAT-3’, reverse, 5’-CACGACTTGTACCACTGGAATCAC-3; IL-6: forward, 5ʹ-TCTCCACAAGCGCCTTCG-3ʹ, reverse, 5ʹ-CTCAGGGCTGAGATGCCG-3ʹ; and 18S: forward, 5’-CCCGGGGAGGTAGTGACGAAAAAT-3’, reverse, 5’-CGCCCGCCCGCTCCCAAGAT-3’. The relative mRNA expression levels of RBMS1 and IL-6 were estimated by the ΔΔCt method and normalized to an 18S internal control.

### Enzyme-linked immunosorbent assay (ELISA)

The protein level of IL-6 derived from the cell culture supernatant was measured by an IL-6 ELISA kit (R&D Systems, MN, USA) according to the manufacturer’s instructions.

### Luminex

Cytokines derived from cell culture supernatants were measured using a human magnetic Luminex assay according to the manufacturer’s instructions (LXSAHM-09hMag Luminex Assay; R&D Systems, Minneapolis, MN, USA).

### Patients and tissue samples

A total of 85 GC tissues and paired adjacent noncancerous gastric formalin-fixed, paraffin-embedded (FFPE) tissues (not less than 20 mm away from GC) specimens surgically resected from primary GC patients were obtained from the First Hospital of China Medical University between Apr 1, 2017 and Sep 31, 2017. Key inclusion criteria: GC patients who have received a radical gastrectomy and D2 node dissection for GC; Key exclusion criteria: patients with secondary malignant tumors. Age, sex, and pathological tumor–node–metastasis (pTNM) stage were evaluated following medical charts and pathology records. pTNM stage was examined according to the Eighth Edition of the American Joint Committee on Cancer (AJCC) cancer staging manual. All research involving human participants was approved by the Ethics Committee of China Medical University (Protocol: AF-SOP-07-1.1-01). Written informed consent was obtained from all the participants in accordance with the Helsinki Declaration.

### Immunohistochemistry (IHC)

The expression of RBMS1 was detected using the IHC staining method. All sections were stained following the protocol of the S-P Immunohistochemical Kit (Zhongshan Jinqiao Biological Technology Ltd., Beijing, China) as previously described (Liu et al., 2020). The staining was evaluated by scanning the entire tissue specimen under low magnification (×10) and confirmed under high magnification (×20 and ×40). Protein expression was visualized and classified based on the percentage of positive cells and the intensity of staining. The heterogeneity of staining was scored as 0 (≤5%), 1 (6–25%), 2 (26–50%), or 3 (>51%). The RBMS1 expression level was calculated based on staining scores of 0, 1, 2, 3, 4, 6, 9, or 12. The final scores were evaluated by two independent pathologists.

### In vivo analysis

All in vivo experiments were performed under the approval of the Institutional Animal Care and Research Committee of China Medical University. Female BALB/c nude mice (8 weeks old) were purchased from Beijing Charles River Laboratory Animal Co., Ltd. and raised in the specific-pathogen-free (SPF) barrier system at China Medical University. The mice were randomly divided into four groups (6 mice per group) and HGC-27-NC and RBMS1-KD, HGC-27-NC, and RBMS1-OE cells (2 × 10^6^/100 µL) were injected via the tail vein to establish a lung metastasis model. The tumor width, length, and body weight of the mice were measured twice per week after injection by investigators who were blinded to the group allocation. The mice were euthanized 8 weeks after injection, and the lung metastatic nodules were counted, collected, and measured for further analysis. Tumor tissues were fixed by 10% formalin and embedded. The sections were stained with hematoxylin and eosin followed by observation under a microscope.

### ^18^F-FDG PET/CT measurements

A PET/CT measurement was performed on each mouse model 8 weeks after GC cells injection. All scans were performed using a small-animal PET scanner (Siemens Healthcare, USA). In all, 10 MBq/0.1–0.2 mL ^18^F-FDG was injected via a tail-vein catheter after anesthetization. PET data were obtained 20 min followed by a delay of 40 min for FDG uptake. In fused PET images, the maximum standardized uptake value (SUV_max_) was calculated from the maximum voxel value (Bq/mL) in the volume of interest.

### Chromatin immunoprecipitation (ChIP) assay

GC cell lines were transfected with siRNAs against RBMS1 and cultured for at least 48 h until at most 90% cell confluency. Immunoprecipitation of sonicated chromatin solutions was conducted by incubation at 4 °C overnight with anti-MYC, anti-RBMS1, and anti-H3K4me3 antibodies. Cross-linking was reversed at 65 °C, and DNA fragments were extracted using phenol–chloroform and precipitated with ethanol. The purified DNA was dissolved in TE buffer and analyzed by regular PCR assay. The results are shown as the percentage of input chromatin, and each experiment was represented from at least three independent experiments. The primers for qPCR were as follows: IL-6: F’: AATAAAGTGCCATGCTGCGA; R’: CAGAATTCCACAG CCTTCCCT; TNFα: F’: GGAAAAGTCAGGGTCTGGAGG; and R’: CCTGGAGGCTC TTTCACTCC.

### Coimmunoprecipitation (Co-IP) analysis

Immunoprecipitation analysis was started with whole-cell lysis purified with anti-IgG for 2 h before interaction. Protein G beads (GE healthcare) concentrated in Sepharose were used for antibody-protein interaction rotation overnight. Western blot assays were performed for further verification.

### Statistical analysis

SPSS version 22.0 was used for statistical analysis. The *χ*^2^ test was used to examine the possible associations between RBMS1 expression and clinicopathological factors. The Kaplan–Meier method was used to evaluate the probability of patient survival. Differences among groups were compared by Student’s *t* test. Each experiment was repeated 3 times, and the data are expressed as mean ± standard deviation. *P* < 0.05 was considered statistically significant.

## Results

### RBMS1 is upregulated in GC cell lines and GC tissues

**Fig. 1:**
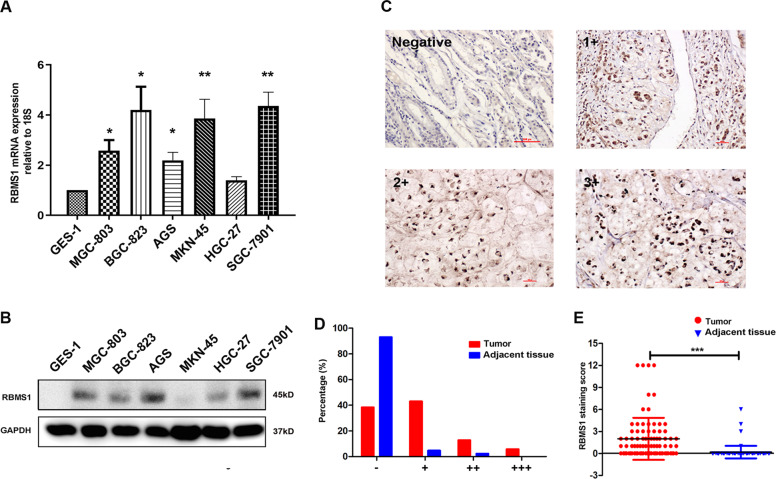
RBMS1 is upregulated in GC tissues and cell lines. **A** RBMS1 mRNA and protein expression (**B**) were detected by RT–PCR and western blot analysis in the GES-1, MGC-803, BGC-823, AGS, MKN-45, HGC-27, and SGC-7901 cell lines. **C** The expression levels of RBMS1 in GC tissues and adjacent GC tissues were measured by IHC staining. The original magnification is ×20. Negative, negative control; **D** Comparison of IHC-0, 1+, 2+, and 3+ percentages between GC tumor and adjacent normal tissues; **E** comparison of RBMS1 staining scores between GC tumor and adjacent normal tissues. *P < 0.05, **P < 0.01, ***P < 0.001.

We first assessed the expression level of RBMS1 in GC cell lines (MGC-803, BGC-823, AGS, MKN-45, HGC-27, and SGC-7901) and normal gastric epithelial cells (GES-1). As shown in Fig. 1A, B, both the mRNA and protein levels of RBMS1 were significantly higher in GC cells than in the GES-1 cell line. Additionally, RBMS1 protein expression was analyzed in patients with resectable GC enrolled from our clinical center in 2017 by IHC staining. As presented in Fig. 1C, cells positively expressing RBMS1 were identified by the presence of brown–yellow particles distributed in the cytoplasm in carcinoma tissue but not in adjacent nonmalignant tissues. The extent of the area of the tumor cells that was stained with brown–yellow particles was assessed as 0, 1+, 2+, or 3+. As presented in Fig. 1D, a total of 37.6% (32/85) of patients were defined as IHC-0, and another 43.5% (37/85), 12.9% (11/85), and 5.9% (5/85) of patients were categorized as 1+, 2+, and 3+, respectively. The RBMS1 IHC staining score strictly followed the scoring system described in the Methods section. As depicted in Fig. 1E, the staining scores were significantly higher in GC tissues than in adjacent normal tissues (*P* < 0.001). Moreover, a high expression level of RBMS1 was positively correlated with stage T3/T4 and positive lymph node metastatic status (Tables S2 and S3). These data indicated that RBMS1 was overexpressed in both GC cell lines and GC tissues.

### RBMS1 overexpression predicts a poor prognosis in GC patients based on public databases

**Fig. 2:**
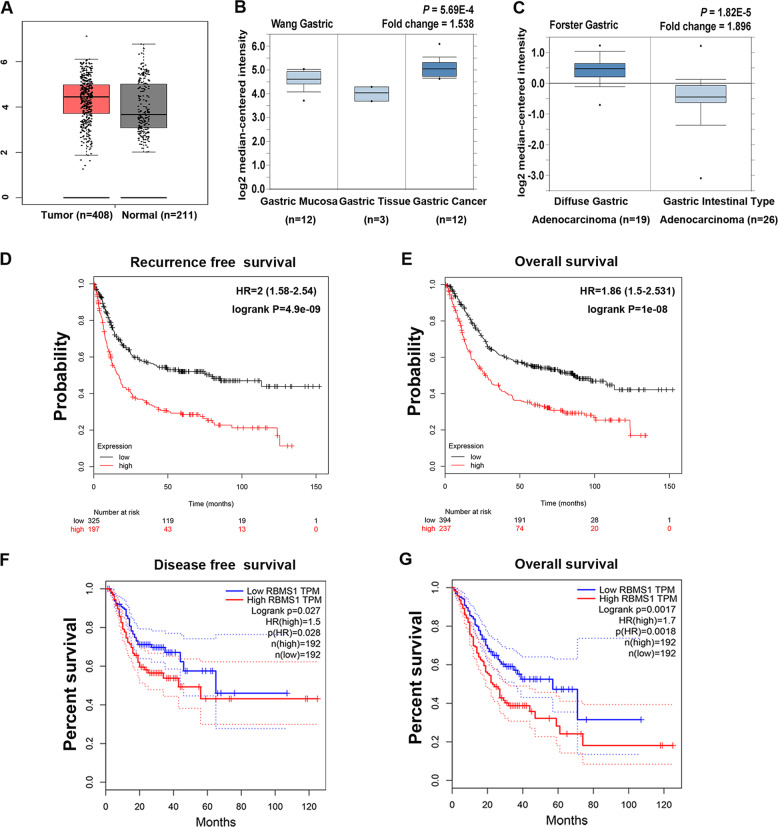
RBMS1 overexpression predicts a poor prognosis in GC patients based on the TCGA and Oncomine databases. **A** Analysis of RBMS1 expression using the Gene Expression Profiling Interactive Analysis (GEPIA) website (http://gepia.cancer-pku.cn/). **B**, **C** Analysis of RBMS1 expression based on the Oncomine database. Analysis of the associations between RBMS1 and recurrence-free survival (**D**) or overall survival (**E**) in GC patients performed via the Kaplan–Meier plotter database. Analysis of the associations between RBMS1 and the disease-free survival (**F**) or overall survival (**G**) of GC patients based on the TCGA database.

To further investigate the role of RBMS1 in the development and progression of GC, we analyzed RBMS1 expression in 408 GC tissues and 211 normal gastric tissues from The Cancer Genome Atlas (TCGA) database. The analysis showed that RBMS1 was significantly upregulated in GC tissue compared with normal tissue (Fig. 2A, *P* < 0.001). Further analysis of the Oncomine database suggested that RBMS1 mRNA expression was significantly elevated in GC tissues compared to gastric tissues and gastric mucosa (Fig. 2B, *P* < 0.001). RBMS1 overexpression was significantly associated with diffuse gastric adenocarcinoma (Fig. 2C, *P* < 0.001). Further analysis of the association between RBMS1 and prognosis in GC patients was performed in the Kaplan–Meier plotter and TCGA databases. The results showed that a higher RBMS1 mRNA expression level was significantly correlated with an increased risk of relapse (HR = 2.0, *P* < 0.001; HR = 1.5, *P* < 0.001, Fig. 2D, F) and mortality (HR = 1.86, *P* < 0.001; HR = 1.7, *P* < 0001, Fig. 2E, G).

**Fig. 3:**
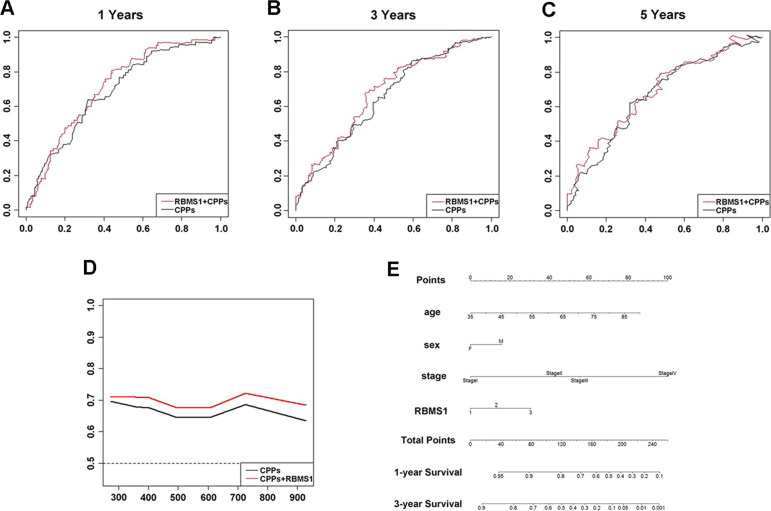
New model combining CPPs with RBMS1 predicts poor clinical outcomes in GC better than CPPs alone. **A**–**C** Calibration plots of the nomogram-predicted and observed 1-, 3- and 5-year survival rates. The nomogram-predicted probability of survival was plotted on the *x*-axis, and actual survival was plotted on the *y*-axis. Dashes: ideal model; vertical bars, 95% confidence interval. **D** Time-dependent receiver operating characteristic (ROC) analysis showed that the combination of RBMS1 with CPPs increased the prediction accuracy of traditional CPPs for overall survival. **E** Nomogram for predicting 1-, 3-, and 5-year survival in GC patients. CPPs, clinicopathological parameters.

These findings were also confirmed based on clinical cohorts from the Oncomine database, which showed that GC patients with high RBMS1 mRNA levels had lower 3-year and 5-year OS rates than those with low RBMS1 mRNA levels (Fig. 3A–C). To provide a quantitative method for better outcome prediction, we constructed a nomogram that integrated several parameters that were suggested to be independent prognostic factors for GC, including age, sex, stage, and RBMS1 (Fig. 3D). The calibration plots of the nomogram indicated that clinicopathological parameters (CPPs) in combination with RBMS1 predicted 1-, 3- and 5-year overall survival rates much better than CPPs alone (Fig. 3E). Taken together, these results indicated that elevated RBMS1 expression was is only involved in carcinogenesis but also predicts poor clinical outcomes in GC.

### RBMS1 promotes the migration and invasion of GC cell lines

**Fig. 4:**
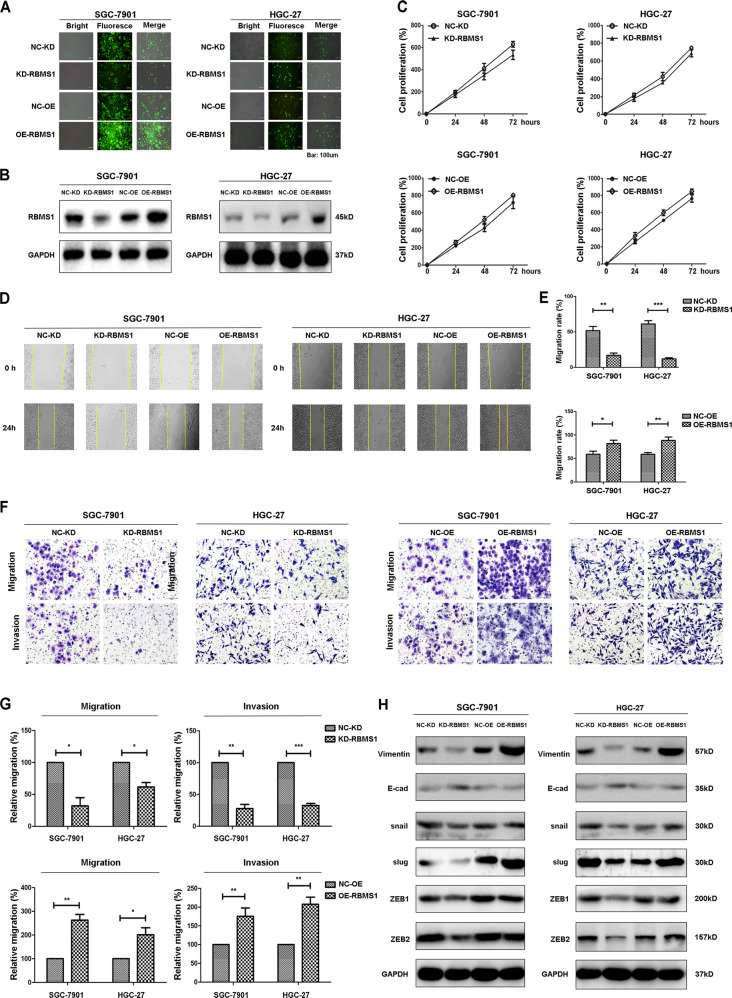
RBMS1 promotes the migration and invasion of GC cells in vitro. **A** SGC-7901 and HGC-27 cells transfected with RBMS1-knockdown and RBMS1-overexpression lentivirus were assessed under a bright-field microscope (left panels: magnification, ×10) and green fluorescence microscope (middle panels: magnification, ×10). Scale bars = 100 μm. **B** The protein expression of RBMS1 in SGC-7901 and HGC-27 cells was examined by western blot analysis after knocking down or overexpressing RBMS1. **C** MTT assay indicated the viability of RBMS1 knockdown or RBMS1-overexpressing SGC-7901 and HGC-27 cells. **D** A wound-healing assay was performed in SGC-7901 and HGC-27 cells with knockdown or overexpression of RBMS1. Representative images at the indicated time points are shown (magnification ×20). **E** Quantitative analysis of the migration area was performed for SGC-7901 and HGC-27 cells using ImageJ software. **F** Representative images of SGC-7901 and HGC-27 cells transfected with control or RBMS1 knockdown or overexpression vectors in migration and invasion assays. Scale bars = 100 μm. **G** Bar graphs show the statistics for cell counts. **H** Western blot assay was performed to detect EMT markers in SGC-7901 and HGC-27 cells with knockdown or overexpression of RBMS1. *P < 0.05, **P < 0.01, ***P < 0.001. NC negative control, KD knockdown, OE overexpression.

To explore the role of RBMS1 in the process of gastric cell migration and invasion, we performed MTT assays, wound healing assays, and Transwell assays with SGC-7901 and HGC-27 cell lines after RBMS1 knockdown or overexpression with lentivirus transfection. The efficiency of RBMS1 knockdown and overexpression exceeded 80%, as confirmed by immunofluorescence (Fig. 4A) and Western blotting (Fig. 4B) of the two cell lines. We first used MTT to detect the effect of silencing or overexpression of RBMS1 on the proliferation of GC cells. However, the proliferation rates did not change in either the RBMS1-OE or RBMS1-KD groups (Fig. 4C). Data from both the wound healing and Transwell migration assays showed that the migration and invasion activity in the RBMS1-KD groups decreased significantly compared with that in the control groups of SGC-7901 cells (*P* < 0.05) and HGC-27 cells (*P* > 0.05). Furthermore, the RBMS1-OE groups showed a significant increase in migration ability compared with the control groups of SGC-7901 cells (*P* < 0.05) and HGC-27 cells (*P* < 0.05) (Fig. 4D–G). As RBMS1 was previously reported to be a suppressor of epithelial-mesenchymal transition (EMT) and metastatic liver colonization in colon cancer cells, we further detected EMT-associated marker expression levels after overexpression and knockdown of RBMS1 in both SGC-7901 and HGC-27 cell lines. The results showed that RBMS1-OE could induce EMT in GC cell lines, and RBMS1-KD significantly reversed this process (Fig. 4H). These results indicated that RBMS1 enhanced both the migration and invasion abilities of GC cell lines by promoting the EMT process.

### RBMS1 promoted GC metastasis in a nude mouse model

**Fig. 5:**
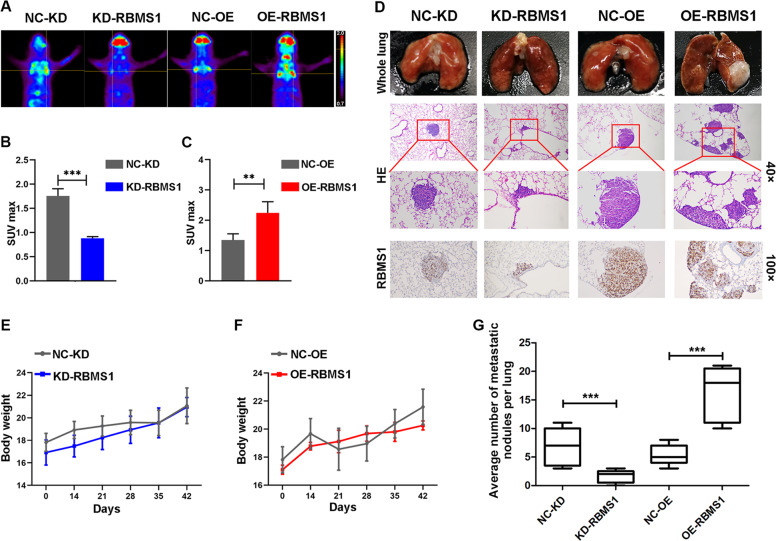
RBMS1 promotes the lung metastasis of GC cells in vivo. RBMS1-NC, RBMS1-KD, and RBMS1-OE HGC-27 cells were injected via the tail vein to establish a lung metastasis mouse model. Small-animal PET/CT scanner was performed for in vivo imaging 8 weeks after injection. Representative PET/CT images were taken (**A**) and SUV_max_ was calculated (**B**, **C**). **D** Mice were killed at 8 weeks after tumor injection to compare the incidence of lung metastasis and tumor weight. Images of a representative lung metastasis mouse model. The IHC staining of the mouse tumor tissues was performed with RBMS1 antibody. Representative images of RBMS1 and H&E staining were shown (magnification ×40 and ×100). **E**, **F** The body weight variation in model mice in all groups was recorded. **G** The number of metastatic lesions in the lung was compared. Lung metastases established from RBMS1-NC cells were considered control groups. Data are mean ± SD for *n* = 6 mice **P < 0.01, ***P < 0.001. NS not significant, NC negative control, KD knockdown, OE overexpression.

To confirm the role of RBMS1 in GC metastasis, an in vivo xenograft model was used. All the mice were randomly grouped and received RBMS1-NC, RBMS1-KD, and RBMS1-OE HGC-27 cells injection via the tail vein to establish a lung metastasis animal model (*n* = 6 per group). A small-animal PET/CT scanner was performed for in vivo imaging 8 weeks after injection. Representative PET/CT images were revealed in Fig. 5A. It was shown that obvious accumulation of FDG was observed in RBMS1-OE mice relative to NC mice and the inhibition of tumor growth was significantly correlated with SUV_max_ (Fig. 5B, C). Moreover, gross inspection showed obviously increased lung metastatic nodules in the RBMS1-OE groups compared with the NC group. In contrast, lung metastasis was significantly inhibited in RBMS1-KD mice relative to NC mice (Fig. 5D). Mouse body weight increased in the RBMS1-OE GC cell injection group but without statistical significance (Fig. 5E, F). Strikingly, the lung lesions derived from RBMS1-OE GC cell injection showed a significantly higher average tumor burden than those derived from NC cells (*P* < 0.01) (Fig. 5G). These results indicated that RBMS1 promoted the growth and metastasis of GC in a nude mouse model.

### IPA revealed potential RBMS1 regulatory mechanisms

**Fig. 6:**
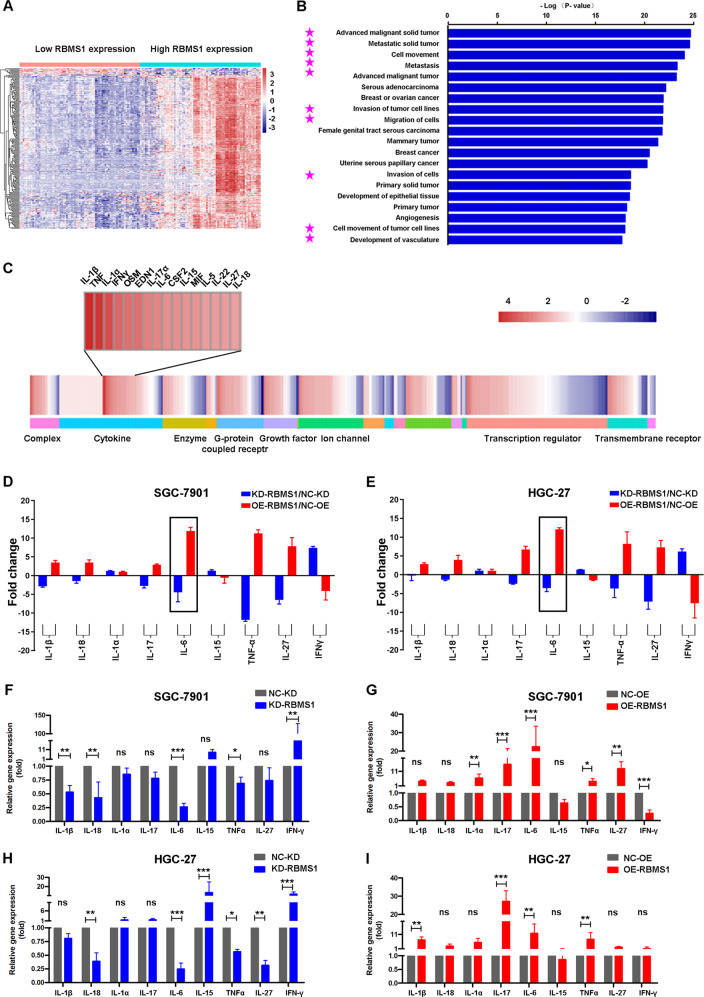
IPA analysis of potential RBMS1 regulatory mechanisms. **A** Heatmap showed the RBMS1-related differentially expressed genes using IPA software. **B** Top 20 affected disease functions based on IPA. The horizontal bars denote the different pathways based on the log (*P* value). Pink stars indicate the metastasis-related functions of cancer. **C** Hierarchical clustering of the DEGs (fold change = 2.0; FDR *P* < 0.05). Each column represents a sample, and each row represents a transcript. The expression level of each gene in a single sample is depicted according to the color scale. **D**, **E** The concentrations of cytokines in culture supernatants of HGC-27 and SGC-7901 cells were measured by Luminex assays after knockdown or overexpression of RBMS1. **F**–**I** The mRNA levels of cytokines were detected by qPCR in the RBMS1-KD and RBMS1-OE GC cell lines. NC negative control, OE overexpression, KD knockdown. NS not significant; *P < 0.05, **P < 0.01, ***P < 0.001.

To further explore the potential regulatory mechanisms of RBMS1 in GC, we analyzed 699 RBMS1-related differentially expressed genes (DEGs) in terms of their network functions, molecular and cellular functions, and canonical signaling pathways using IPA software (Fig. 6A and Table S4). Figure 6B depicts the top 20 significant molecular and cellular functional categories that were associated with RBMS1. Of note, a highly connected network of candidate proteins associated with RBMS1 was analyzed by the IPA algorithm to predict which upstream regulators might be the key factors in the regulation of RBMS1 expression. As presented in Fig. 6C, cytokines were predicted to be the most significant upstream regulators in the upregulated RBMS1 gene set. To further verify the IPA results, we performed qPCR to compare the levels of multiple cytokines before and after overexpression or knockdown of RBMS1 in GC cell lines. Additionally, the concentrations of cytokines in culture supernatants derived from HGC-27 and SGC-7901 cells were measured by Luminex assays. The results showed that IL-6 was obviously upregulated in culture supernatants of RBMS1-OE GC cells (Fig. 6D, E) and at the mRNA level (Fig. 6F–I). The above data analysis results suggested that RBMS1 might promote the migration and invasion of GC cells by regulating autocrine IL-6 expression.

### RBMS1 promotes the migration and invasion of GC cells through autocrine IL-6

**Fig. 7:**
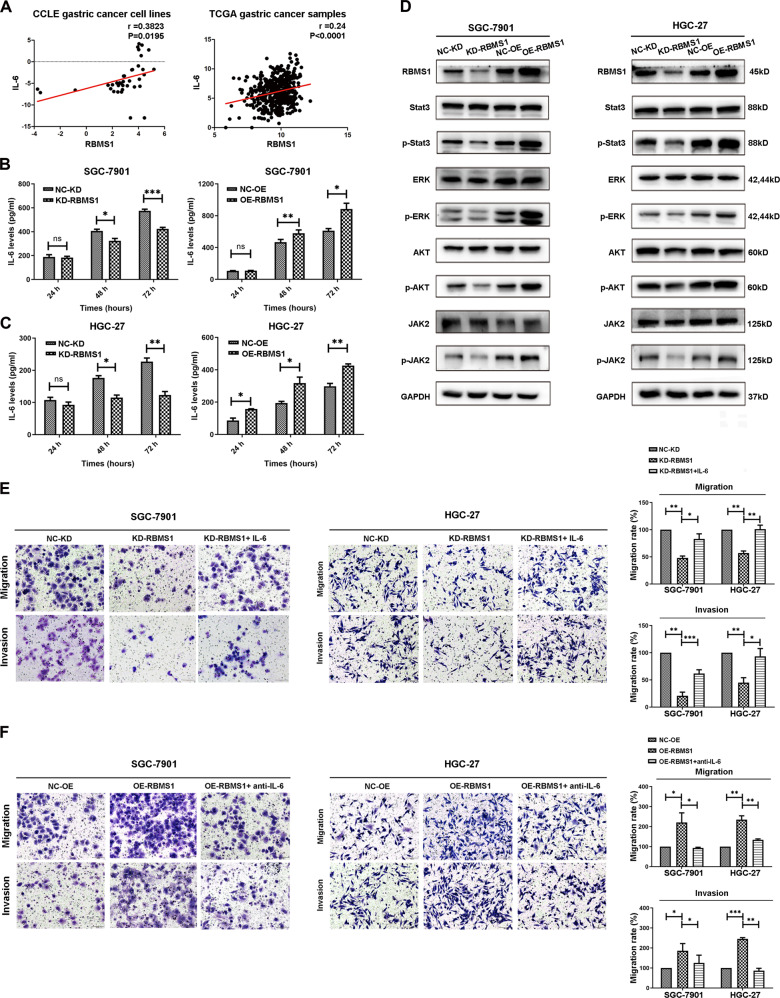
RBMS1 promotes the migration and invasion of GC cells through autocrine IL-6/JAK2/STAT3 signaling. **A** The correlation between RBMS1 and IL-6 in GC in the CCLE and TCGA databases. **B** ELISA showed the change in IL-6 expression after knocking down or overexpressing RBMS1 in SGC-7901 (**B**) and HGC-27 cells (**C**). **D** Western blot analysis indicated the differential levels of STAT3, p-STAT3, ERK, p-ERK, AKT, p-AKT, JAK2, and p-JAK2 after knocking down or overexpressing RBMS1 in SGC-7901 and HGC-27 cells. GAPDH was used as a loading control in the western blot analysis. **E** Transwell assays showed the change in migration and invasion after adding IL-6 to SGC-7901 and HGC-27 cells with RBMS1 knockdown. Scale bars = 100 μm. **F** Transwell assays showed the changes in migration and invasion after IL-6 neutralizing antibody treatment in SGC-7901 and HGC-27 cells overexpressing RBMS1. Scale bars = 100 μm. *P < 0.05, **P < 0.01, ***P < 0.001. NC negative control, KD knockdown, OE overexpression; magnification, ×20.

According to the data analysis results, IL-6 is a potential downstream target of RBMS1. To determine whether RBMS1 regulates the migration and invasion of GC cell lines through autocrine IL-6 signaling, GC cell lines from the Cancer Cell Line Encyclopedia (CCLE) and GC patient samples from the TCGA were used to analyze the relationship between IL-6 and RBMS1 (Fig. 7A). IL-6 and RBMS1 were positively correlated in GC cell lines with a correlation coefficient of 0.3823 (*P* = 0.0195) and in TCGA GC patient samples with a correlation coefficient of 0.24 (*P* < 0.0001). Furthermore, the IL-6 concentration in culture supernatants at different time points after RBMS1 knockdown in HGC-27 and SGC-7901 cells was measured by ELISA, and inhibition of RBMS1 significantly reduced IL-6 protein levels in the cell culture medium (Fig. 7B, C). Conversely, overexpression of RBMS1 increased IL-6 protein levels in the cell culture medium (Fig. 7B, C). Western blot assays further indicated that the phosphorylation of classical downstream targets of IL-6, Janus kinase 2 (JAK2), and signal transducer and activator of transcription 3 (STAT3) was also significantly downregulated following the knockdown of RBMS1 expression; overexpression of RBMS1 upregulated the phosphorylation of JAK2/STAT3 signaling; however, the total protein level did not change significantly (Fig. 7D). Then, recombinant human IL-6 or IL-6 neutralizing antibody was added to detect the migration and invasion abilities of GC cells after knocking down or overexpressing RBMS1. Treatment with IL-6 significantly reversed the inhibitory effect of RBMS1 knockdown on the migration and invasion abilities of GC cells (Fig. 7E). Conversely, the addition of an IL-6 neutralizing antibody to RBMS-overexpressing GC cells significantly inhibited migration and invasion (Fig. 7F). Overall, these results indicated that RBMS1 promotes the migration and invasion of GC cells through autocrine IL-6/JAK2/STAT3 signaling.

### RBMS1 interacts with MYC to regulate cytokine transactivation

**Fig. 8:**
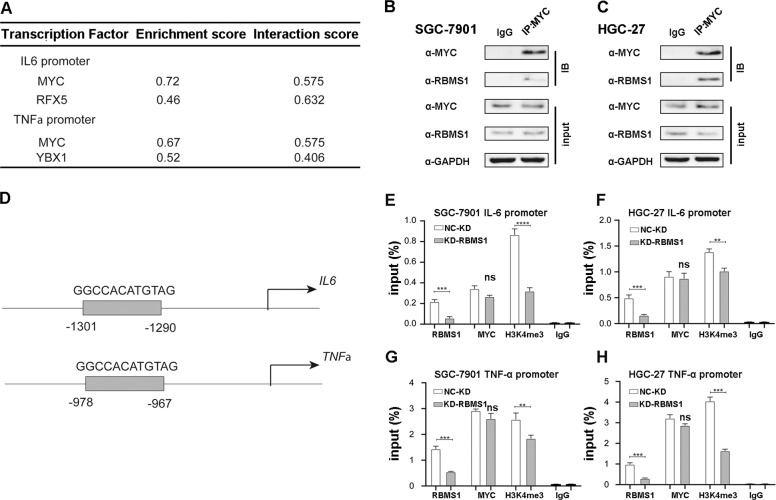
RBMS1 interacts with MYC to regulate cytokine transactivation. **A** The three-line table shows the recruitment and binding coefficients of transcription factors interacting with RBMS1 in the two gene promoter regions. Enrichment scores were formed by the GeneHancer website, and interaction scores were obtained from the STRING website. **B**, **C** Co-IP assay showed the interaction between RBMS1 and MYC in SGC-7901 (**B**) and HGC-27 (**C**) cells. Input represents 5% of the total cell extract used for each immunoprecipitation; **D** Potential binding sites and sequences of IL-6 and TNF-α promoter MYC predicted from NCBI website; **E**–**H** ChIP assay was performed using the indicated antibodies in two kinds of cells with knocking down RBMS1 expression by siRBMS1 and analyzed by qPCR. The data are representative of more than two independent experiments. **P < 0.01, ***P < 0.001, ****P < 0.0001. NS not significant, NC negative control, KD knockdown, OE overexpression.

As RBMS1 is one of the MYC gene single-stranded binding proteins and has been demonstrated to control proto-oncogene c-MYC expression in human cells, we hypothesized that RBMS1 may regulate cytokine expression after binding with MYC. To further clarify the molecular mechanism, we chose two cytokine targets, IL6 and TNFα, that changed simultaneously after the overexpression of RBMS1. Utilizing the GeneHancer database, we screened the promoter regions of IL6 and TNFα for all potential transcription factors that regulated both genes. According to the Search Tool for the Retrieval of Interacting Genes/Proteins (STRING) database, we observed that RBMS1 might regulate IL6 and TNFα through the transcription factor MYC (Fig. 8A). Subsequently, protein immunoprecipitation (Co-IP) assays were performed with both SGC-7901 and HGC-27 cells. The results showed that RBMS1 could interact with MYC in both GC cell lines (Fig. 8B, C). We used the NCBI website and found the conceivable binding sites of MYC in the promoters of IL6 and TNFα and designed ChIP primers (Fig. 8D). As detected in ChIP assays, knockdown of RBMS1 essentially decreased the H3K4me3 modification of regulatory elements of two genes but did not impact MYC protein recruitment (Fig. 8E–H). These results indicated that RBMS1 impacts IL6 transactivation by influencing histone modification in the promoter regions after binding with MYC. However, this mechanism needs further investigation in future experiments.

## Discussion

GC is one of the most prominent malignant tumors, and metastasis is a complex multistep process that requires the regulation of many cellular pathways and functions. In the present study, we revealed the important role of RBMS1 as a candidate promoter gene for GC metastasis via analysis of a high-throughput sequencing dataset and clinical specimens and explored a prognostic model for the estimation of tumor-associated mortality in GC patients. Most importantly, we shed light on the potential molecular mechanisms by which RBMS1 promotes GC metastasis: it transactivates IL-6 and stimulates the JAK2/STAT3 downstream signaling pathway based on both in vitro and in vivo experiments, which suggests that RBMS1 may be a potential therapeutic target for GC.

Although many studies over the past several years have demonstrated that multiple genetic alterations of tumor suppressors and tumor-associated genes are responsible for the metastasis process of GC [22], in most cases, the contribution of this information to the improvement of overall survival is still small. It is not evident which genetic pathway leads to the aggressive phenotype as a key element in GC. The challenge is to detect stage-specific genetic abnormalities that may aid in early diagnosis and even aid in selecting effective therapeutic strategies in GC. Therefore, in the present study, we extracted gene expression data from 408 GC patients from the GOBO and Oncomine databases and identified one core gene, RBMS1, that was closely associated with an aggressive phenotype and a poor prognosis in GC.

The RBMS1 gene encodes a member of a small family of proteins that have been implicated in diverse functions, such as DNA replication, gene transcription, cell cycle progression, and apoptosis coordinated by c-Myc, a major proto-oncogene in human cancer [23, 24]. It has been reported to bind to both single- and double-stranded sequences of a putative DNA replication origin sequence in the human c-Myc oncogene [9, 25]. Furthermore, RBMS1 stimulates c-Myc-derived apoptosis induction and cell transformation by binding to c-Myc [26]. However, the biological functions and molecular mechanisms of RBMS1 in carcinogenesis remain to be elucidated. In this study, bioinformatics was used to explore RBMS1 expression via public databases containing GC data. According to the meta-analysis of two datasets, overexpression of RBMS1 was detected in most diffuse and mixed gastric adenocarcinoma tissues and associated with poor prognosis. We also confirmed that RBMS1 expression was obviously increased in GC tissues compared with corresponding adjacent noncancerous tissues by IHC staining based on GC patient tissues collected from our clinical cancer center. These results were consistent with the bioinformatics analysis results, which highlighted the same trends in GC. Moreover, we performed a series of functional assays and observed that RBMS1 significantly promoted the migration and invasion of GC cells by promoting the EMT process in vitro and in vivo. Taken together, these results strongly indicated that RBMS1 may play a critical role in GC metastasis as a core gene.

However, to date, there have been very few studies on the biological function of RBMS1 in GC. To explore the molecular mechanism of RBMS1-induced migration in GC, highly connected pathways of the “core interactome” in the core gene set and RBMS1-related DEGs were analyzed using the IPA algorithm, and these DEGs were highly enriched in pathways associated with carcinogenesis, metastasis, and cell movement. It is worth noting that we further screened for possible downstream regulators of RBMS1, and IL-6 might be a potential downstream regulator. Many studies have reported that IL-6 is highly upregulated in several types of cancers and is considered one of the most important cytokines during cancer development and metastasis [27]. It has been demonstrated that IL-6 is capable of the EMT process induction in various types of cancer [28]. Additionally, high levels of circulating IL-6 have been reported to be positively correlated with cancer metastasis [29]. However, whether RBMS1 could regulate IL-6 expression and promote GC metastasis is still unclear. In our study, we validated this hypothesis by performing intracellular experiments and analyzing tissue specimens. IL-6 protein and mRNA levels were reduced both in the supernatant and in GC cells after RBMS1 silencing and overexpression based on Luminex and qPCR assays. These results suggested that RBMS1 might promote the migration and invasion of GC cells through IL-6.

In line with this, to functionally characterize RBMS1 in the regulation of IL-6 in GC metastasis, we screened the promoter regions of IL6 for all potential transcription factors utilizing the GeneHancer database and identified MYC as the most likely transcription factor. After demonstrating that RBMS1 could interact with MYC in both GC cell lines, we speculated that RBMS1 might regulate IL6 transactivation through MYC based on STRING database analysis. It has been reported that H3K4me3 on promoter regions is usually associated with gene activation. Hence, we performed ChIP assays to assess the recruitment of associated proteins that might be impacted by RBMS1 with an anti-H3K4me3 antibody. Our work demonstrated that RBMS1 could upregulate IL-6 transactivation by influencing histone modification in promoter regions and then induce autocrine IL-6/JAK2/STAT3 signaling to facilitate GC metastasis. However, further experiments are needed to clarify the detailed molecular mechanisms. In addition, due to the limited follow-up time and sample size of this study, a study involving a larger sample size and long-term follow-up may be needed in the future to assess the characteristics related to RBMS1 in GC patients.

In conclusion, we provided compelling evidence that RBMS1 is upregulated and predicts a poor prognosis and poor clinical outcomes in GC patients. We also elucidated the critical role of RBMS1 in the promotion of GC metastasis: it acts by inducing autocrine IL-6/JAK2/STAT3 signaling and the EMT process; in addition, RBMS1 upregulates IL-6 expression and secretion by IL6 transactivation by influencing histone modification in the promoter regions after binding with the transcription factor MYC. Our novel findings ultimately indicate that RBMS1 can be considered a potential prognostic or therapeutic biomarker. The precise molecular mechanism deserves further investigation.

## Supplementary information

Equest agreement from all authors

Table S1. Antibody resources table

Table S2. Clinicopathological characteristics of 85 patients with gastric cancer.

Table S3. Associations between RBMS1 expression and clinicopathological characteristics

Table S4. Signaling pathways analyzed by IPA

AJ-checklist

Language editing certification

## Data Availability

The data in the current study are available from the corresponding authors upon reasonable request.
