# Supplementary material for: RBMS1 promotes gastric cancer metastasis through autocrine IL-6/JAK2/STAT3 signaling
Source: Cell Death Dis. 2022 Mar 31;13(3):287. doi: 10.1038/s41419-022-04747-3 (PMC8971453; doi:10.1038/s41419-022-04747-3)
Supplement: Supplementary file 2 — Table S1. Antibody resources table [file 41419_2022_4747_MOESM2_ESM.docx]

| **Antibodies** | **Clone** | **Source** | **Dilution** | **分子量(kD)** |
| --- | --- | --- | --- | --- |
| Anti- Rabbit RBMS1(WB) | Ab51344 | Abcam | 1:1000 | 45 |
| Anti- Rabbit RBMS1(IHC) | LS-C405789 | LSBio | 1:30 |  |
| Anti-Rabbit GAPDH | FL-335 | Santa | 1:1000 | 37 |
| Anti- Rabbit STAT3 | 79D7 | CST | 1:2000 | 84,91 |
| Anti- Rabbit p-STAT3 | Tyr705 | CST | 1:500 | 79,86 |
| Anti- Rabbit ERK | 137F5 | CST | 1:1000 | 42,44 |
| Anti- Rabbit p-ERK | D13.14.4E | CST | 1:1000 | 42,44 |
| Anti- Rabbit AKT | C67E7 | CST | 1:1000 | 60 |
| Anti- Rabbit p-AKT | D9E | CST | 1:500 | 60 |
| Anti- Rabbit JAK2 | D2E12 | CST | 1:1000 | 125 |
| Anti- Rabbit p-JAK2 | Tyr1007/1008 | CST | 1:1000 | 125 |
| Anti- Rabbit E-cadherin | 24E10 | CST | 1:1000 | 135 |
| Anti- Rabbit Vimentin | D21H3 | CST | 1:1000 | 57 |
| Anti- Rabbit Snail | C15D3 | CST | 1:1000 | 30 |
| Anti- Rabbit Slug | C19G7 | CST | 1:1000 | 30 |
| Anti- Rabbit ZEB1 | D80D3 | CST | 1:1000 | 200 |
| Anti-Mouse ZEB2 | E-11 | Santa | 1:250 | 157 |
| Secondary goat anti-rabbit antibody | Goat anti-rabbit IgG-HRP sc-2004 | Santa | 1:2000 |  |
| Secondary goat anti-mouse antibody | Goat anti-mouse IgG-HRP sc-2005 | Santa | 1:2000 |  |

**Table S1. Antibody resources table**
