# Supplementary material for: RBMS1 promotes gastric cancer metastasis through autocrine IL-6/JAK2/STAT3 signaling
Source: Cell Death Dis. 2022 Mar 31;13(3):287. doi: 10.1038/s41419-022-04747-3 (PMC8971453; doi:10.1038/s41419-022-04747-3)
Supplement: Supplementary file 3 — Table S2. Clinicopathological characteristics of 85 patients with gastric cancer. [file 41419_2022_4747_MOESM3_ESM.doc]

Table S2. Clinicopathological characteristics of 85 patients with gastric cancer.

| Characteristics | Cases（%） |
| --- | --- |
| Age(year) |  |
| < 59 | 17（20.0%） |
| ≥ 60 | 68（80.0%） |
| Sex |  |
| Female | 36（42.4%） |
| Male | 49（57.6%） |
| Histological grade |  |
| G1 | 10 （11.7%） |
| G2 | 19 （22.3%） |
| G3 | 56 （66.0%） |
| T stage |  |
| T1+T2 | 30（35.3%） |
| T3+T4 | 55（64.7%） |
| pN stage |  |
| N0 | 37（43.5%） |
| N1+2+3 | 48（56.5%） |
