# Supplementary material for: RBMS1 promotes gastric cancer metastasis through autocrine IL-6/JAK2/STAT3 signaling
Source: Cell Death Dis. 2022 Mar 31;13(3):287. doi: 10.1038/s41419-022-04747-3 (PMC8971453; doi:10.1038/s41419-022-04747-3)
Supplement: Supplementary file 4 — Table S3. Associations between RBMS1 expression and clinicopathological characteristics [file 41419_2022_4747_MOESM4_ESM.doc]

Table S3. Associations between RBMS1 expression and clinicopathological characteristics

| **Characteristics** | **Cases** | **RBMS1 expression in GC** | | |
| --- | --- | --- | --- | --- |
|  |  | **Low (%)** | **High (%)** | **P value** |
| **Age(year)** |  |  |  | 0.742 |
| < 59 | 17 | 6 (35.3) | 11(57.6) |  |
| ≥ 60 | 68 | 27(39.7) | 41(60.3) |  |
| **Age** |  |  |  | 0.318 |
| Male | 49 | 25(51.0) | 24(49.0) |  |
| Female | 36 | 18(50.0) | 18(50.0) |  |
| **Histological grade** |  |  |  | 0.881 |
| G1 | 10 | 1(10.0) | 9(90.0) |  |
| G2 | 19 | 11(57.9) | 8(42.1) |  |
| G3 | 56 | 21(37.5) | 35(62.5) |  |
| **Tumor stage** |  |  |  | 0.012 |
| T1+2 | 30 | 17(56.7) | 13(43.3) |  |
| T3+4 | 55 | 18(32.7) | 37(67.3) |  |
| **pN stage** |  |  |  | <0.0001 |
| N0 | 37 | 22(59.5) | 15(40.5) |  |
| N1+2+3 | 48 | 11(22.9) | 37(77.1) |  |
