# Supplementary material for: RBMS1 promotes gastric cancer metastasis through autocrine IL-6/JAK2/STAT3 signaling
Source: Cell Death Dis. 2022 Mar 31;13(3):287. doi: 10.1038/s41419-022-04747-3 (PMC8971453; doi:10.1038/s41419-022-04747-3)
Supplement: Supplementary file 5 — Table S4. Signaling pathways analyzed by IPA [file 41419_2022_4747_MOESM5_ESM.docx]

**Table S4. Signaling pathways analyzed by IPA**

| Ingenuity Canonical Pathways | -log(p-value) | Ratio | z-score |
| --- | --- | --- | --- |
| IL-6 Signaling | 2.75 | 0.0355 | 2.646 |
| Colorectal Cancer Metastasis Signaling | 2.2 | 0.0282 | 2.646 |
| Osteoarthritis Pathway | 3.98 | 0.0427 | 2.333 |
| G Beta Gamma Signaling | 2.13 | 0.0455 | 2 |
| HMGB1 Signaling | 2.94 | 0.0451 | 1.633 |
| IL-8 Signaling | 2.27 | 0.0391 | 1.342 |
| Dendritic Cell Maturation | 2.14 | 0.0311 | 1.342 |
| cAMP-mediated signaling | 1.82 | 0.0264 | 1.342 |
