# Supplementary material for: RBMS1 promotes gastric cancer metastasis through autocrine IL-6/JAK2/STAT3 signaling
Source: Cell Death Dis. 2022 Mar 31;13(3):287. doi: 10.1038/s41419-022-04747-3 (PMC8971453; doi:10.1038/s41419-022-04747-3)
Supplement: Supplementary file 7 — Language editing certification [file 41419_2022_4747_MOESM7_ESM.pdf]

SPRINGER NATURE

# Author Services

## Editing Certificate

This document certifies that the manuscript

**RBMS1 promotes gastric cancer metastasis through autocrine IL-6/JAK2/STAT3 signaling**

prepared by the authors

Mengyuan Liu, Huijing Zhang, Huan Zhou, Taiwei Jiao, Mingliang Feng, Mingjun Sun, Lei Xue, Lu Xu

was edited for proper English language, grammar, punctuation, spelling, and overall style by one or more of the highly qualified native English speaking editors at SNAS.

This certificate was issued on **December 13, 2021** and may be verified on the SNAS website using the verification code **5599-AE9C-8128-9560-4B5P** .

Neither the research content nor the authors' intentions were altered in any way during the editing process. Documents receiving this certification should be English-ready for publication; however, the author has the ability to accept or reject our suggestions and changes. To verify the final SNAS edited version, please visit our verification page at secure.authorservices.springernature.com/certificate/verify. If you have any questions or concerns about this edited document, please contact SNAS at support@as.springernature.com.

SNAS provides a range of editing, translation, and manuscript services for researchers and publishers around the world.
For more information about our company, services, and partner discounts, please visit authorservices.springernature.com.
