# Supplementary material for: RBMS1 promotes gastric cancer metastasis through autocrine IL-6/JAK2/STAT3 signaling
Source: Cell Death Dis. 2022 Mar 31;13(3):287. doi: 10.1038/s41419-022-04747-3 (PMC8971453; doi:10.1038/s41419-022-04747-3)
Supplement: Supplementary file 1 — Equest agreement from all authors [file 41419_2022_4747_MOESM1_ESM.pdf]

回复: Notification to co-authors of submission to Cell Death & Disease CDDIS-21-1939RR

发件人：liheming8563<liheming8563@hotmail.com>
时　间：2022年3月17日(星期四) 上午10:41
收件人：徐璐<xulu@cmu.edu.cn>

Hi Dr. Xu

Thank you for your email. I agree.

Best

Heming Li

从 Windows 版邮件发送

发件人: 徐璐
发送时间: 2022年3月17日 10:25
收件人: liheming8563
主题: Notification to co-authors of submission to Cell Death & Disease CDDIS-21-1939RR

Dear Dr. Li
You are receiving this email because you have been listed as the first author (equal contribution) on a manuscript recently submitted to Cell Death & Disease (CDDIS-21-1939RR). The manuscript details are below.
Title: RBMS1 promotes gastric cancer metastasis through autocrine IL-6/JAK2/STAT3 signaling
Authors: Mengyuan Liu#, Heming Li#, Huijing Zhang, Huan Zhou, Taiwei Jiao, Mingliang Feng, Fangjian Na, Mingjun Sun, Mingfang Zhao, Lei Xue*, Lu Xu*
Corresponding authors: Lei Xue and Lu Xu
If you are agree with this, please reply.
Kind regards,

Lu Xu
Department of Medical Oncology,
The First Hospital of China Medical University
Email: xulu@cmu.edu.cn

Re:Notification to co-authors of submission to Cell Death & Disease CDDIS-21-1939RR

发件人：zmf<zhaomf618@126.com>
时 间：2022年3月17日(星期四) 上午10:42
收件人：徐璐<xulu@cmu.edu.cn>

Dear Dr. Xu

I agree.

Thank you

Mingfang Zhao

--

Mingfang Zhao, M.D.
Department of Medical Oncology
the First Hospital of China Medical University
Shenyang, 110001
P.R. China
Email: zhaomf618@126.com

At 2022-03-17 10:32:41, "徐璐" <xulu@cmu.edu.cn> wrote:

> Dear Dr. Zhao
>
> You are receiving this email because you have been listed as the co-author on a manuscript recently submitted to Cell Death & Disease (CDDIS-21-1939RR). The manuscript details are below.
>
> Title: RBMS1 promotes gastric cancer metastasis through autocrine IL-6/JAK2/STAT3 signaling
>
> Authors: Mengyuan Liu#, Heming Li#, Huijing Zhang, Huan Zhou, Taiwei Jiao, Mingliang Feng, Fangjian Na, Mingjun Sun, Mingfang Zhao, Lei Xue*, Lu Xu*
>
> Corresponding authors: Lei Xue and Lu Xu
>
> If you are agree with this, please reply.
>
> Kind regards,
>
> Lu Xu
> Department of Medical Oncology,
> The First Hospital of China Medical University
> Email: xulu@cmu.edu.cn

回复：Notification to co-authors of submission to Cell Death & Disease CDDIS-21-1939RR

发件人：xuelei4970362<xuelei4970362@sina.com>
时　间：2022年3月17日(星期四) 上午10:47
收件人：徐璐<xulu@cmu.edu.cn>

Dear Dr. Xu
Thank you for adding my name. I totally agree.
Our study is very interesting and thank you all for the contribution.

Lei Xue

----- 原始邮件 -----
发件人："徐璐" <xulu@cmu.edu.cn>
收件人："xuelei4970362" <xuelei4970362@sina.com>
主题：Notification to co-authors of submission to Cell Death & Disease CDDIS-21-1939RR
日期：2022年03月17日 10点36分

Dear Dr. Xue
You are receiving this email because you have been listed as the corresponding authors on a manuscript recently submitted to Cell Death & Disease (CDDIS-21-1939RR). The manuscript details are below.
Title: RBMS1 promotes gastric cancer metastasis through autocrine IL-6/JAK2/STAT3 signaling
Authors: Mengyuan Liu#, Heming Li#, Huijing Zhang, Huan Zhou, Taiwei Jiao, Mingliang Feng, Fangjian Na, Mingjun Sun, Mingfang Zhao, Lei Xue*, Lu Xu*
Corresponding authors: Lei Xue and Lu Xu
If you are agree with this, please reply.
Kind regards,

Lu Xu
Department of Medical Oncology,
The First Hospital of China Medical University
Email: xulu@cmu.edu.cn

Re:Notification to co-authors of submission to Cell Death & Disease CDDIS-21-1939RR

发件人：歌曲<najerry@126.com>
时　间：2022年3月17日(星期四) 上午10:50
收件人：徐璐<xulu@cmu.edu.cn>

Dear Dr. Xu

Thank you for your email. I agree and am willing to be an co-author on a manuscript recently submitted to Cell Death & Disease (CDDIS-21-1939RR).

Kind regards,

Fangjian Na
Network Information Center,
China Medical University
Email:najerry@126.com

At 2022-03-17 10:37:52, "徐璐" <xulu@cmu.edu.cn> wrote:

> Dear Dr. Na
>
> You are receiving this email because you have been listed as the co-authors on a manuscript recently submitted to Cell Death & Disease (CDDIS-21-1939RR). The manuscript details are below.
>
> Title: RBMS1 promotes gastric cancer metastasis through autocrine IL-6/JAK2/STAT3 signaling
>
> Authors: Mengyuan Liu#, Heming Li#, Huijing Zhang, Huan Zhou, Taiwei Jiao, Mingliang Feng, Fangjian Na, Mingjun Sun, Mingfang Zhao, Lei Xue*, Lu Xu*
>
> Corresponding authors: Lei Xue and Lu Xu
>
> If you are agree with this, please reply.
>
> Kind regards,
>
> Lu Xu
> Department of Medical Oncology,
> The First Hospital of China Medical University
> Email: xulu@cmu.edu.cn
